# Effects of oceanographic environment on the distribution and migration of Pacific saury (*Cololabis saira*) during main fishing season

**DOI:** 10.1038/s41598-022-17786-9

**Published:** 2022-08-09

**Authors:** Shigang Liu, Yang Liu, Jianchao Li, Chang Cao, Hao Tian, Wenjia Li, Yongjun Tian, Yoshiro Watanabe, Longshan Lin, Yuan Li

**Affiliations:** 1 Frontiers Science Center for Deep Ocean Multispheres and Earth System and Key Laboratory of Mariculture, Ministry of Education, Ocean University of China, Qingdao, China; 2 Third Institute of Oceanography, Ministry of Natural Resources, Xiamen, China; 3 Laboratory for Marine Fisheries Science and Food Production Processes, Pilot National Laboratory for Marine Science and Technology, Qingdao, China; 4 Atmosphere and Ocean Research Institute, University of Tokyo, Tokyo, Japan

**Keywords:** Zoology, Ecology, Environmental sciences

## Abstract

The Pacific saury (*Cololabis saira*) is one of the most commercially important pelagic fishes in Asia–Pacific countries. The oceanographic environment, especially the Oyashio Current, significantly affects the distribution of Pacific saury, and may lead to variations in their migration route and the formation of fishing grounds in Japanese coastal region and the high seas. In this study, six oceanographic factors, sea surface temperature (SST), sea surface chlorophyll-*a* concentration (SSC), sea surface salinity (SSS), sea surface height (SSH), mixed layer depth (MLD), and eddy kinetic energy (EKE), were associated with the monthly catch per unit effort 1 (monthly CPUE_1_, ton/vessel) and the monthly CPUE_2_ (ton/day) of Pacific saury from Chinese fishing vessels during the optimal fishing periods (September–November) in 2014–2017. The gradient forest analysis showed that the performance of monthly CPUE_1_ was higher than monthly CPUE_2_ and SST was the most important oceanographic factor influencing monthly CPUE_1_, followed by EKE. The generalized additive model indicated that SST, SSH, and EKE negatively affected monthly CPUE_1_, whereas SSC, SSS, and MLD induced dome-shaped increases in monthly CPUE_1_. The distributions of fishing locations are likely to form along Offshore Oyashio current and meanders, especially in October and November. Synchronous trends in the relationship between the intrusion area of the Oyashio and relative abundance variation index suggest that an increase in the intrusion area of the Oyashio causes more Pacific saury to migrate to the Japanese coastal region, and vice versa. These findings extend our understanding of the effects of the oceanographic environment on Pacific saury.

## Introduction

The Pacific saury (*Cololabis saira*) is one of the most commercially important small pelagic species in Japan, Chinese Taipei, China, Korea, Russia and Vanuatu, and is widely distributed throughout the middle latitudes of the North Pacific^1–4^. Pacific saury lifespans are 1–2 years, and they reach sexual maturity in about 280 days^5,6^. The spawning period of Pacific saury extends from September to June, with individual fish spawning several times during the single extended spawning season^7,8^.

**Figure 1:**
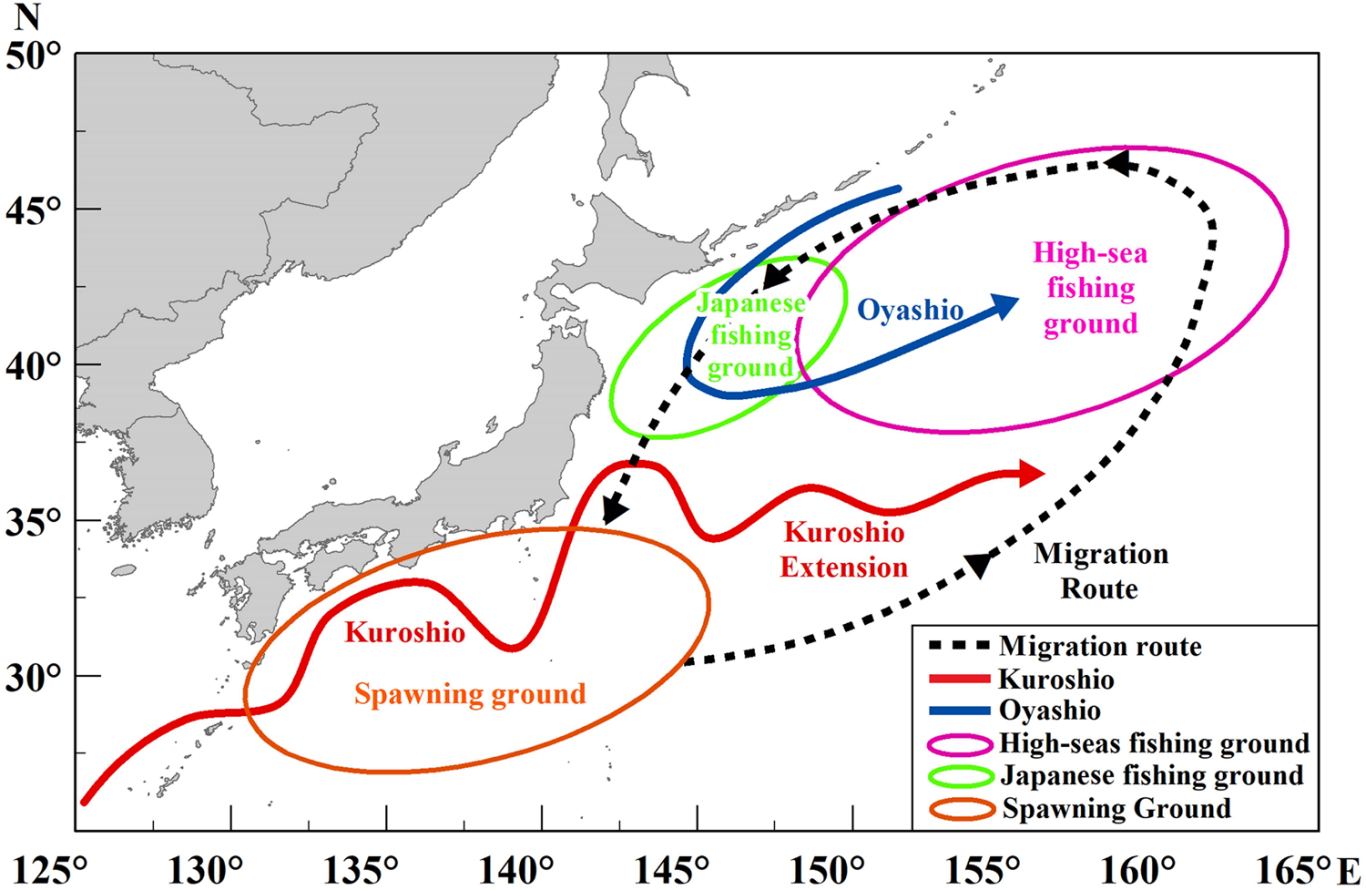
Schematic diagram of the spawning ground, fishing grounds, and migration route of Pacific saury, with the main oceanographic structures along the Pacific coast of Japan. Solid red line represents the Kuroshio Current and Kuroshio Extension, and the solid blue line represents the Oyashio. Dotted line with arrows represents the migration route of Pacific saury. Orange ellipse indicates the Kuroshio region, the main spawning area of Pacific saury in winter. Green ellipse indicates the Japanese fishing ground, and violet ellipse indicates the high-sea fishing ground. Map were created using ArcGIS 10.2 sofware by Esri (www.arcgis.com).

The optimal fishing period for Pacific saury is autumn (September–November), when they migrate south^8^. The catch and catch per unit effort (CPUE) are higher in autumn than in other months^4^. Fisheries in Japan and Russia have long Pacific saury histories, and their fishing grounds are mainly located within their exclusive economic zones (EEZs), whereas China, Chinese Taipei, and Korea harvest the Pacific saury mainly in the high-sea fishing grounds to the east of Japan and Russia’s EEZs^9^ (Fig. 1). In recent years, catches of Pacific saury in the northwestern Pacific have greatly declined. The Japanese catch dropped from 354,727 tons in 2008 to 29,562 tons in 2020 (NPFC, https://www.npfc.int/summary-footprint-pacific-saury-fisheries). However, the decline in catch from high-sea fishing grounds was less dramatic, even as catches great decrease in Japanese coastal fishing grounds. This phenomenon has attracted considerable attention, in particular the mechanisms underlying the variation in the abundance and distribution of Pacific saury^10^.

The population dynamics of Pacific saury are more affected by oceanic and climatic factors than by fishing, and annual variations in Pacific saury abundance are strongly associated with changes in winter SST in the Kuroshio Current and the mixed layer depth (MLD) in the Kuroshio Extension^3,11–14^. The mechanisms controlling the regime shifts in the abundance of Pacific saury have been previously well established: changes in the sizes and positions of the spawning ground alter the conditions of spawning and fish survival, which then affect the recruitment and abundance of this species^10^.

The catch of Pacific saury is closely related not only to its recruitment in the Kuroshio spawning ground, but also to the distribution of the Oyashio feeding ground^15,16^. Pacific saury migrates between the subtropical Kuroshio region and the subarctic Oyashio region throughout the Kuroshio–Oyashio Transition Zone, which contains complex oceanic structures^11,17^. Its migration is driven by the requirement for suitable water temperatures for spawning and the need for optimal access to food resources^18,19^. The migratory pattern of Pacific saury is associated with oceanic environmental conditions, such as sea surface temperature (SST), sea surface chlorophyll-*a* concentration (SSC), sea surface salinity (SSS), and sea surface height (SSH)^20–22^. Ocean currents, describing by eddy kinetic energy (EKE), have been shown to particularly affect the distributions of fishing grounds in some species, such as the Japanese common squid (*Todarodes pacificus*) and the skipjack tuna (*Katsuwonus pelamis*)^23,24^. The intensity and direction of the Oyashio significantly affects the coastal Pacific saury fishing ground in Japan^16^. Furthermore, Kakehi et al.^25^ used an ocean circulation model to predict the location of the nearshore migration of Pacific saury^25^. The interactions between the Kuroshio and Oyashio form complex current patterns in the northwestern Pacific^15^, such as the Kuroshio Extension, Offshore Oyashio current, and meso-scale eddies. These current structures determine the changes and the spatial distributions of SST, SSC, and other hydrological characteristics. Therefore, the oceanographic environment, especially the Oyashio, plays an important role in the distribution of Pacific saury. These effects may cause variations in the migration route of Pacific saury, thus affecting the formation of fishing grounds in Japanese coastal region and the high seas.

To accurately predict the catch and distribution of Pacific saury, research needs to concentrate on the oceanographic environment, which significantly affects this species. Compared with numerous studies of the coastal fishing grounds, our understanding of the migration and distribution of Pacific saury in the high seas is limited, and only a few studies have focused on the relationships underlying the variations of catch between coastal and high seas^4,9,16^. In this study we combined gradient forest analysis and generalized additive model, and used the SST, SSC, SSS, SSH, MLD, and EKE as indicators in the model. The purpose of this study is to examine the importance and effects of the oceanographic environment on the distribution of Pacific saury in high-sea fishing ground during main fishing season, and to clarify the significant impact of the Oyashio and eddies on the distribution of the species, in order to explain the variations in its catch in coastal and high-sea fishing grounds.

## Results

### The monthly variation in the distributions of Pacific saury caused by the changes in the oceanographic environments

#### Temporal–spatial distribution

**Figure 2:**
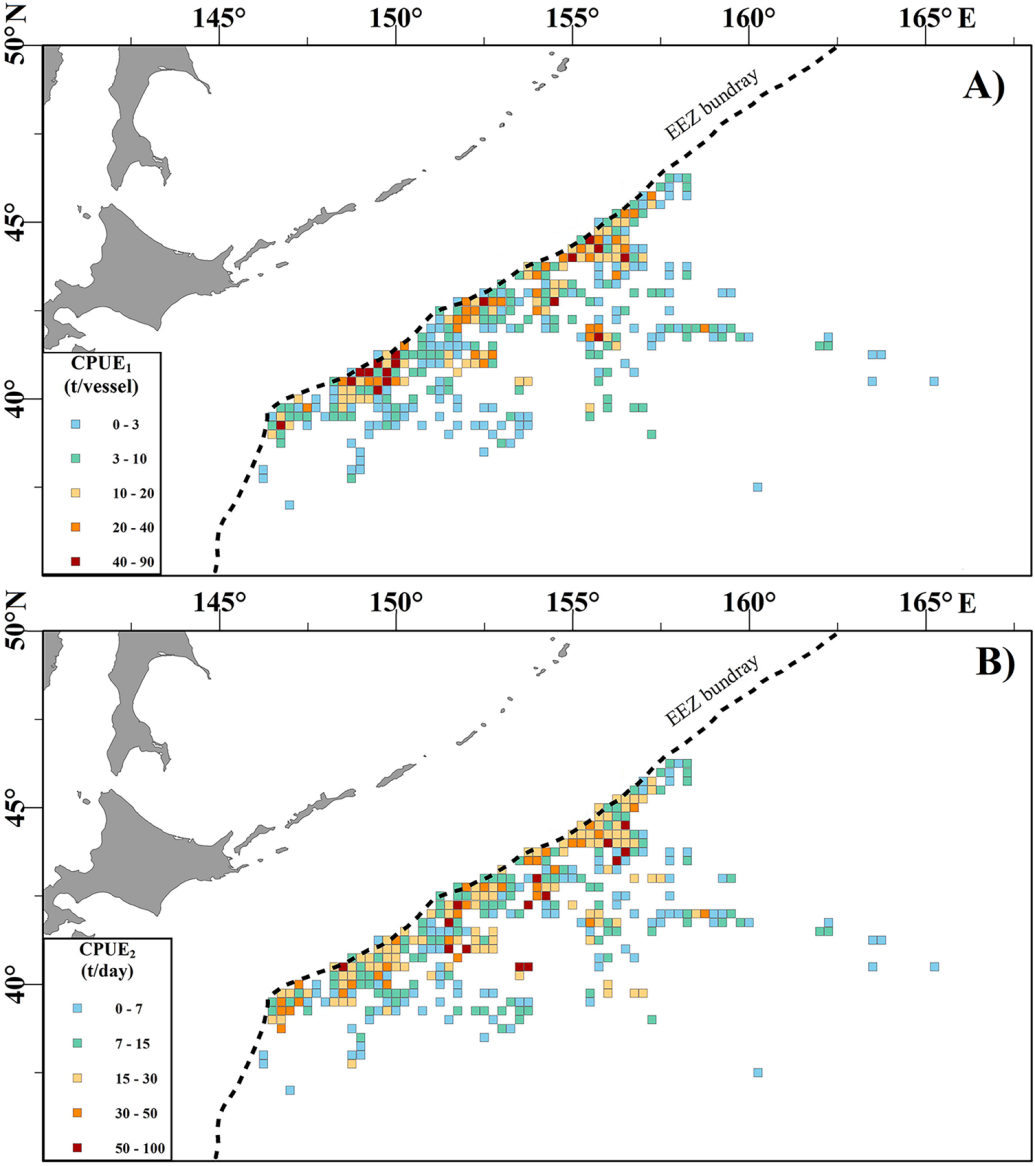
Spatial distributions of (**A**) annual average CPUE_1_ and (**B**) CPUE_2_ for Pacific saury of Chinese stick-held dip net fishing vessels in the northwestern Pacific Ocean in 2014–2017. The black dotted line is the EEZ boundary. Maps were created using ArcGIS 10.2 sofware by Esri (www.arcgis.com).

The main fishing ground for fishing vessels in the high seas was at 38–47° N, 146–160° E during September–November (Fig. 2). Most fishing locations were concentrated in the western part of the high-sea fishing ground, close to the Japanese EEZ. The grids with higher catches were predominantly distributed along Japan’s EEZ, except for the grids around 42° N, 156° E. monthly CPUE_1_ and CPUE_2_ were higher along the coast of Japan. However, the grids with the highest monthly CPUE_2_ were more dispersed and more easterly than the maximum monthly CPUE_1_ grids, thus the distribution patterns of monthly CPUE_1_ were not entirely the same those of monthly CPUE_2_.

**Figure 3:**
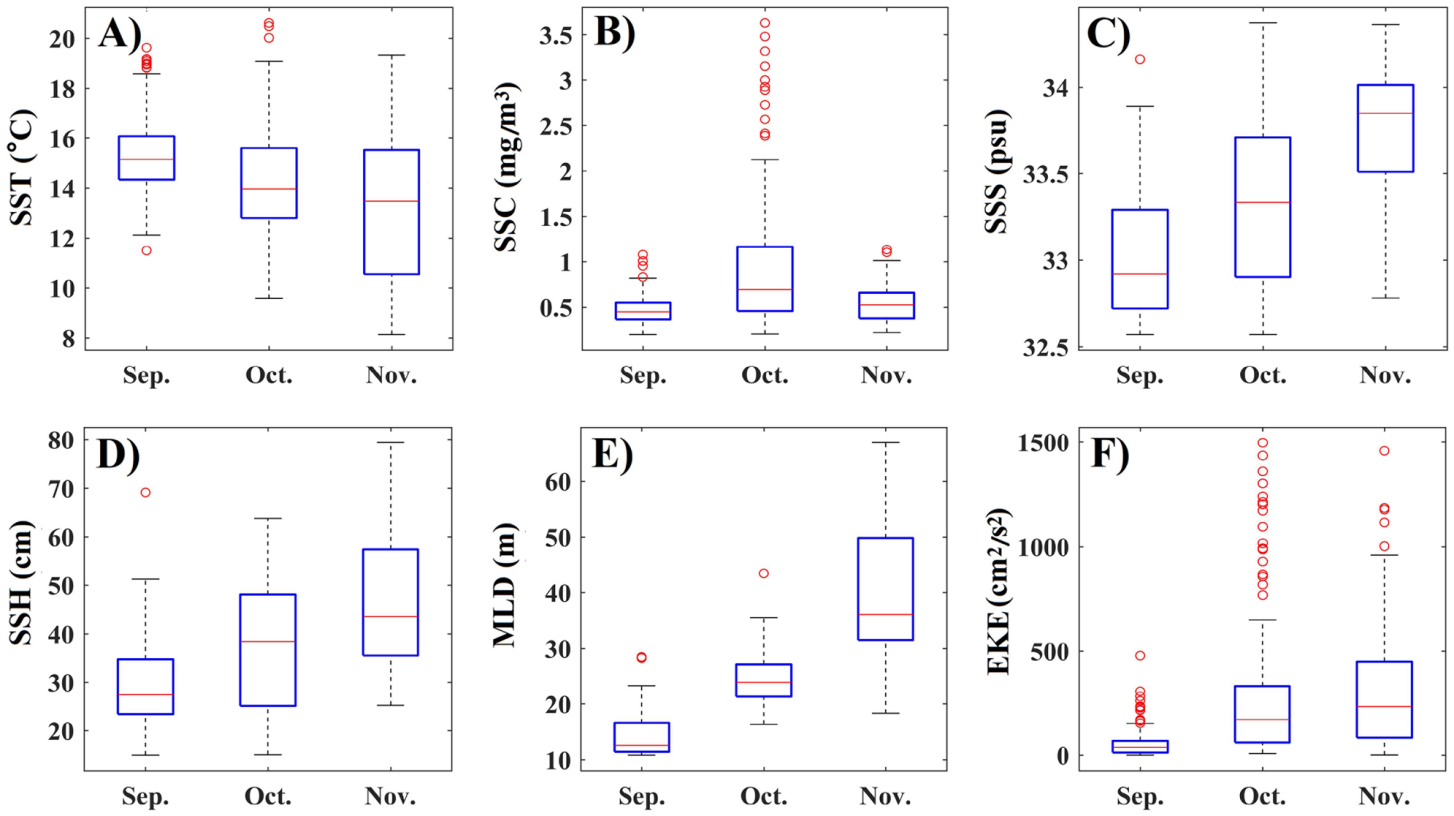
Box plot of environmental variables affecting Pacific saury in the northwestern Pacific in September–November between 2014 and 2017: (**A**) SST; (**B**) SSC; (**C**) SSS; (**D**) SSH; (**E**) MLD; and (**F**) EKE.

**Figure 4:**
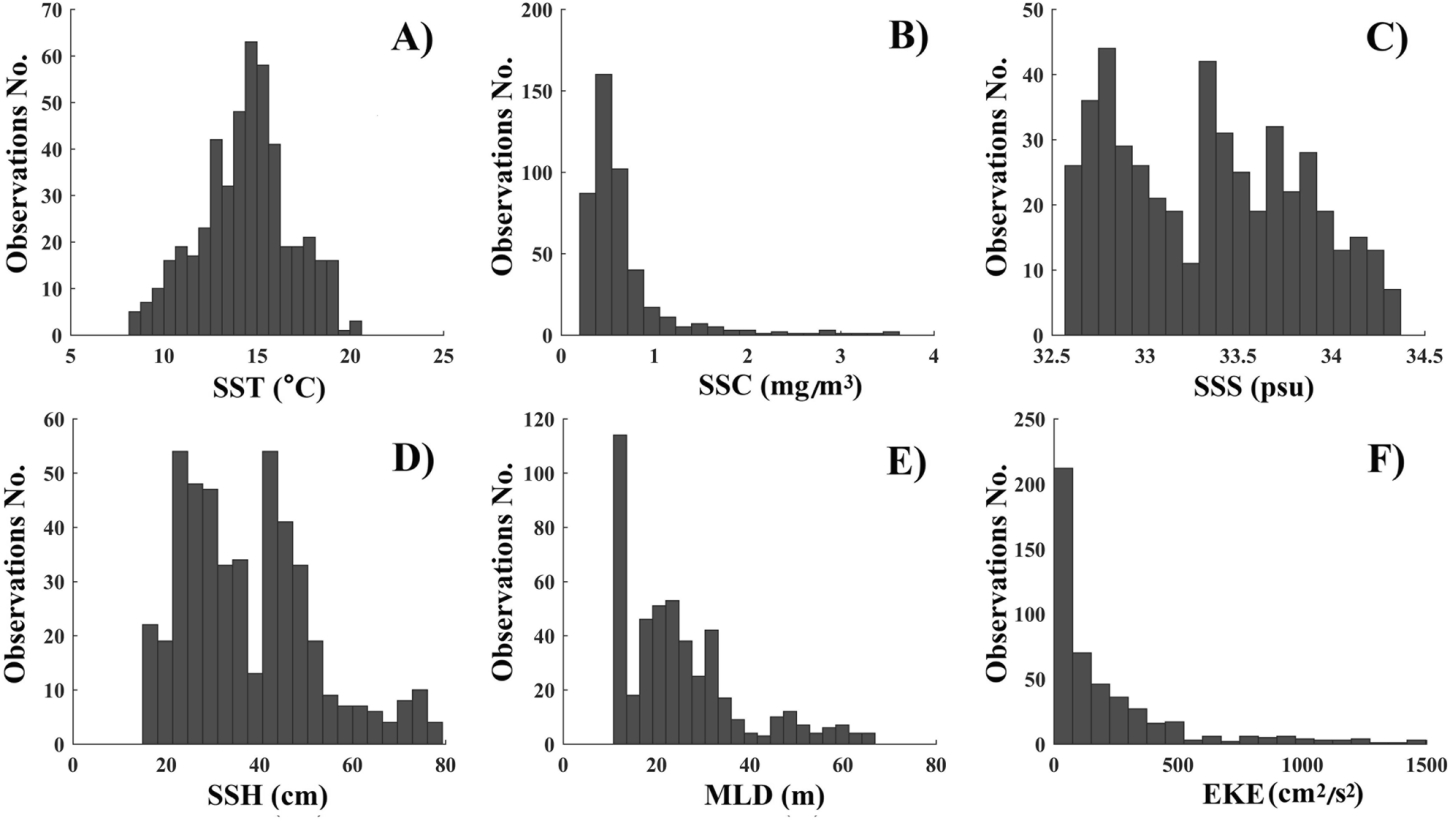
Histograms of environmental variables affecting Pacific saury in the northwestern Pacific in September–November between 2014 and 2017: (**A**) SST; (**B**) SSC; (**C**) SSS; (**D**) SSH; (**E**) MLD; and (**F**) EKE.

The monthly fishing locations were superimposed on corresponding images of the oceanographic environment (Fig. S1). The variations in monthly environmental factors at the fishing locations showed different trends (Fig. 3). In September–November, SST tended to decrease gradually, whereas SSS, SSH, MLD, and EKE tended to increase gradually, while SSC were highest in October. In fishing locations, SST varied from 8.1 to 20.6 °C, with a peak around 15 °C; SSC varied from 0.2 to 3.6 mg/m^3^, with a peak around 0.5 mg/m^3^; SSS varied from 32.5 to 34.3 psu, with two peaks around 32.7 and 33.4 psu; SSH varied from 14.9 to 79.3 cm, with two peaks around 0.23 and 0.41 cm; MLD varied from 10.8 to 66.9 m, with two peaks around 12 and 22 m; EKE varied from 0.2 to 1496.2 cm^2^/s^2^, with a peak around 35 cm^2^/s^2^ (Fig. 4).

#### Importance of environmental factors

**Figure 5:**
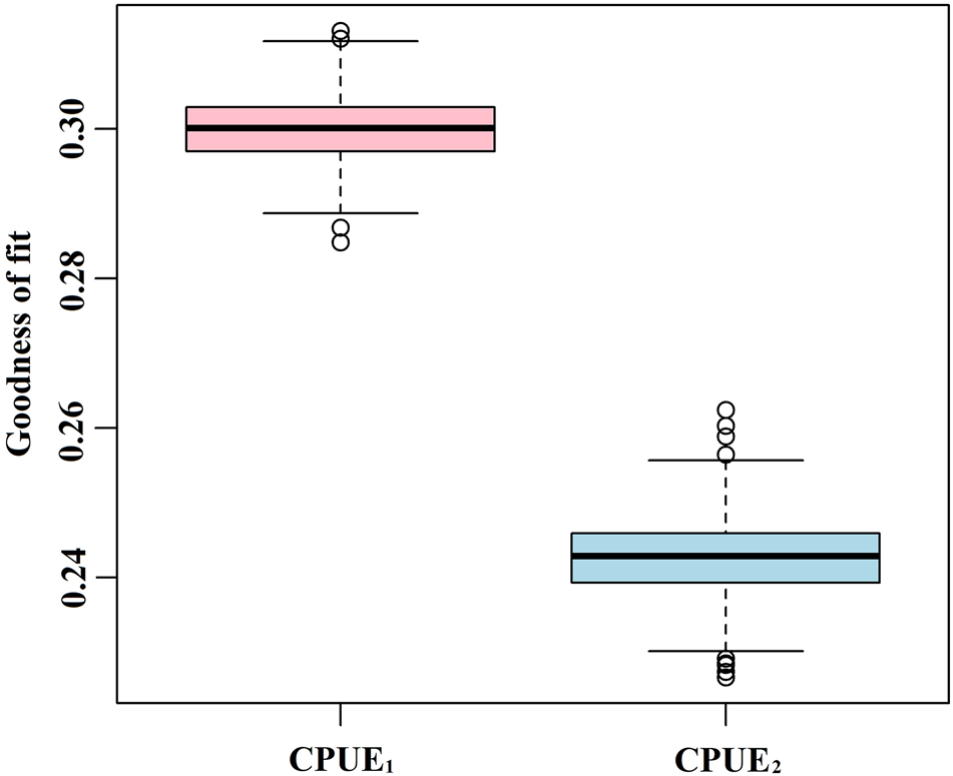
Model performance (goodness of fit, R^2^) of 1000 runs of the gradient forest analysis for CPUE_1_ and CPUE_2_.

**Figure 6:**
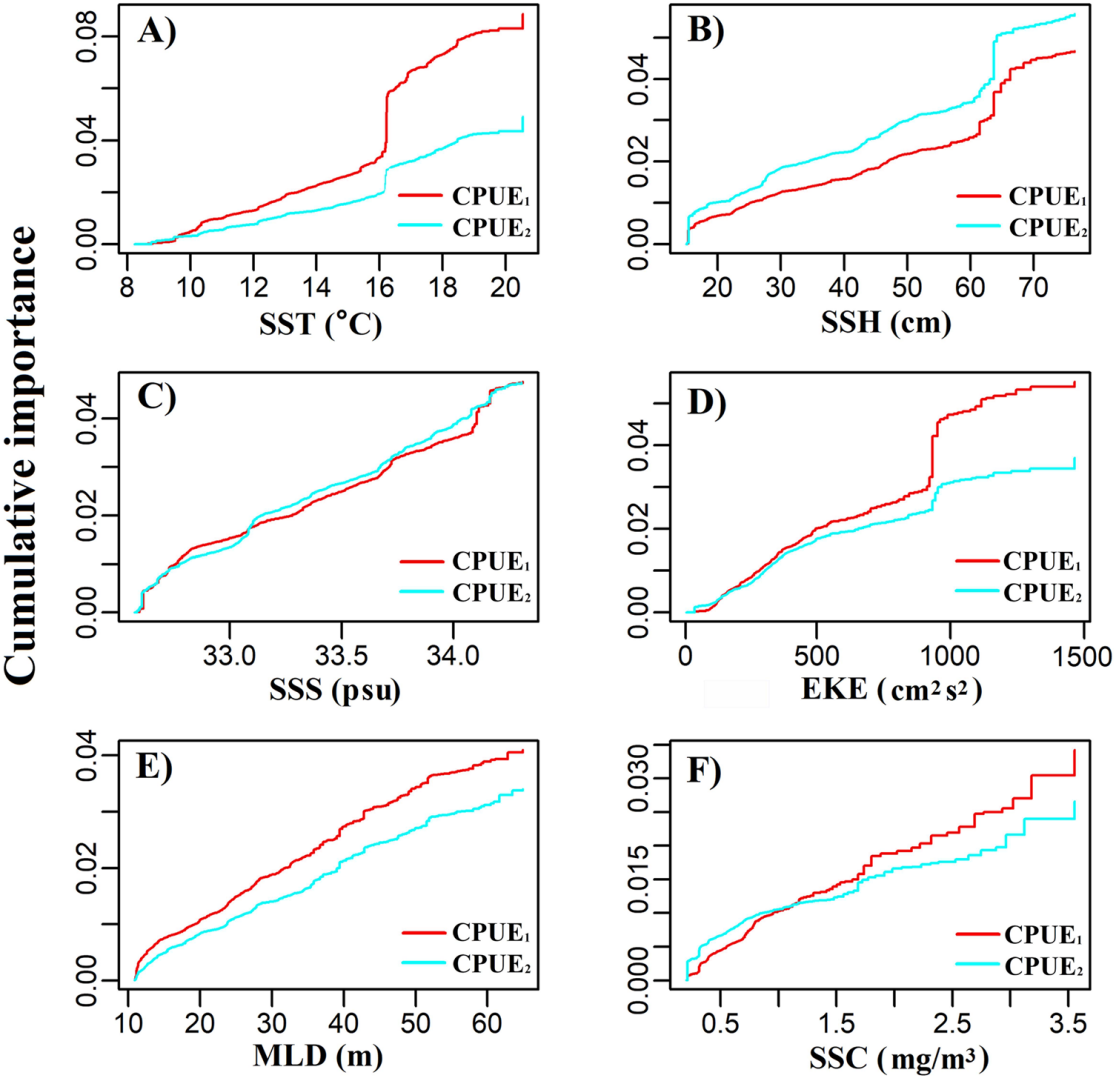
Cumulative shifts (in R^2^ units) in CPUE_1_ and CPUE_2_ in response to environmental variables (SST, SSC, SSS, SSH, MLD, and EKE).

Based on 1000 runs of gradient forest analyses, the performance (goodness of fit, ${R}_{f}^{2}$) of monthly CPUE_1_ (mean = 0.300, *sd* = 0.004) was significantly higher than that of monthly CPUE_2_ (mean = 0.242, *sd* = 0.005), indicating that environmental factors predict monthly CPUE_1_ better than they predict monthly CPUE_2_ (Fig. 5). Of the 1000 runs, the one with the best performance (highest *R*^2^) was used to quantify the relationship between each environmental factor and the monthly CPUE_1_ of Pacific saury. We weighted the responses of monthly CPUE_1_ and CPUE_2_ for each environmental factor, which was calculated as the cumulative importance distribution of the split improvement scaled by the R^2^-weighted importance and standardized by the density of observations (Fig. 6). The monthly CPUE_1_ and CPUE_2_ for Pacific saury had strong threshold responses when SST, SSH, and EKE were around 16.1 °C, 65 cm, and 900 cm^2^/s^2^, respectively. The strong threshold response indicated a better fit between monthly CPUE and environmental factors. For monthly CPUE_1_, SST, EKE, MLD, and SSC showed greater cumulative importance, whereas for monthly CPUE_2_, SSH showed greater cumulative importance. The cumulative importance of SSS was approximately the same for monthly CPUE_1_ and CPUE_2_.

**Figure 7:**
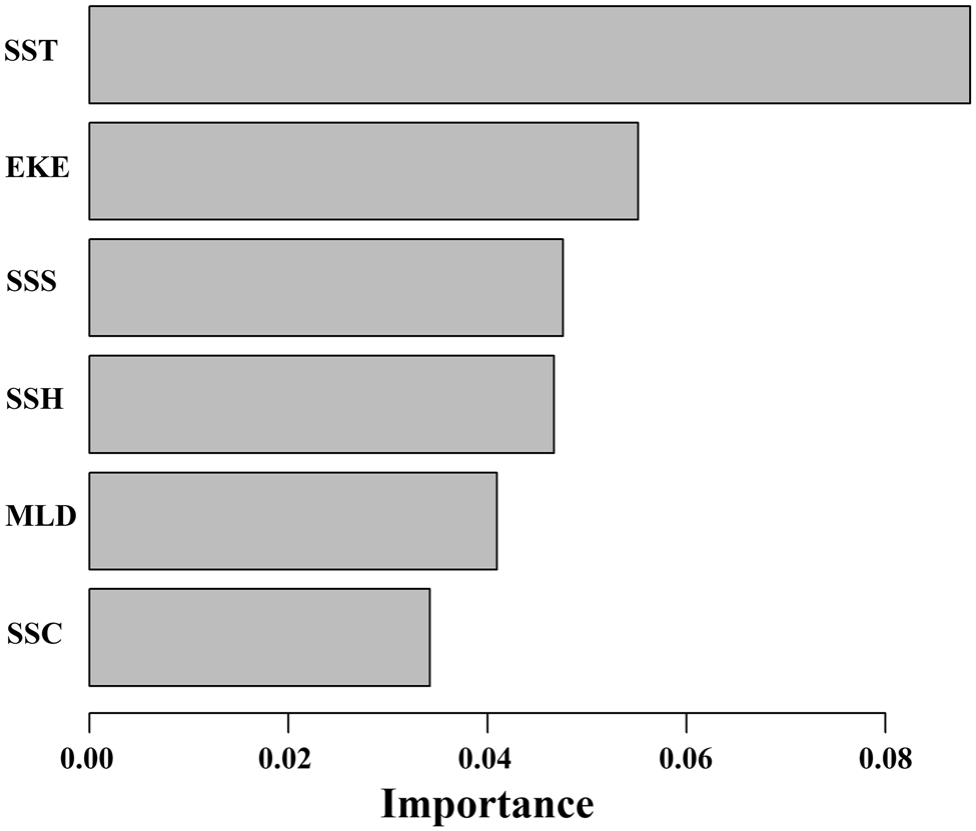
Importance of environmental variables (SST, SSC, SSS, SSH, MLD, and EKE) across CPUE_1_ outputs (R^2^) according to gradient forest analyses.

Environmental factors more strongly affected the monthly CPUE_1_ of Pacific saury than the monthly CPUE_2_, and we therefore only calculated the importance of each environmental factor for monthly CPUE_1_ as the response variable in the gradient forest analyses. The mean importance of the pressure variables (R^2^), measured by their contribution to the prediction accuracy of the OOB response, was 0.034–0.089. The most important predictor was SST (mean importance R^2^ = 0.089), measured as its contribution to the prediction accuracy for the OOB samples, followed by EKE (R^2^ = 0.055), SSS (R^2^ = 0.047), SSH (R^2^ = 0.046), MLD (R^2^ = 0.041), and SSC (R^2^ = 0.034) (Fig. 7).

#### Influence trends

**Figure 8:**
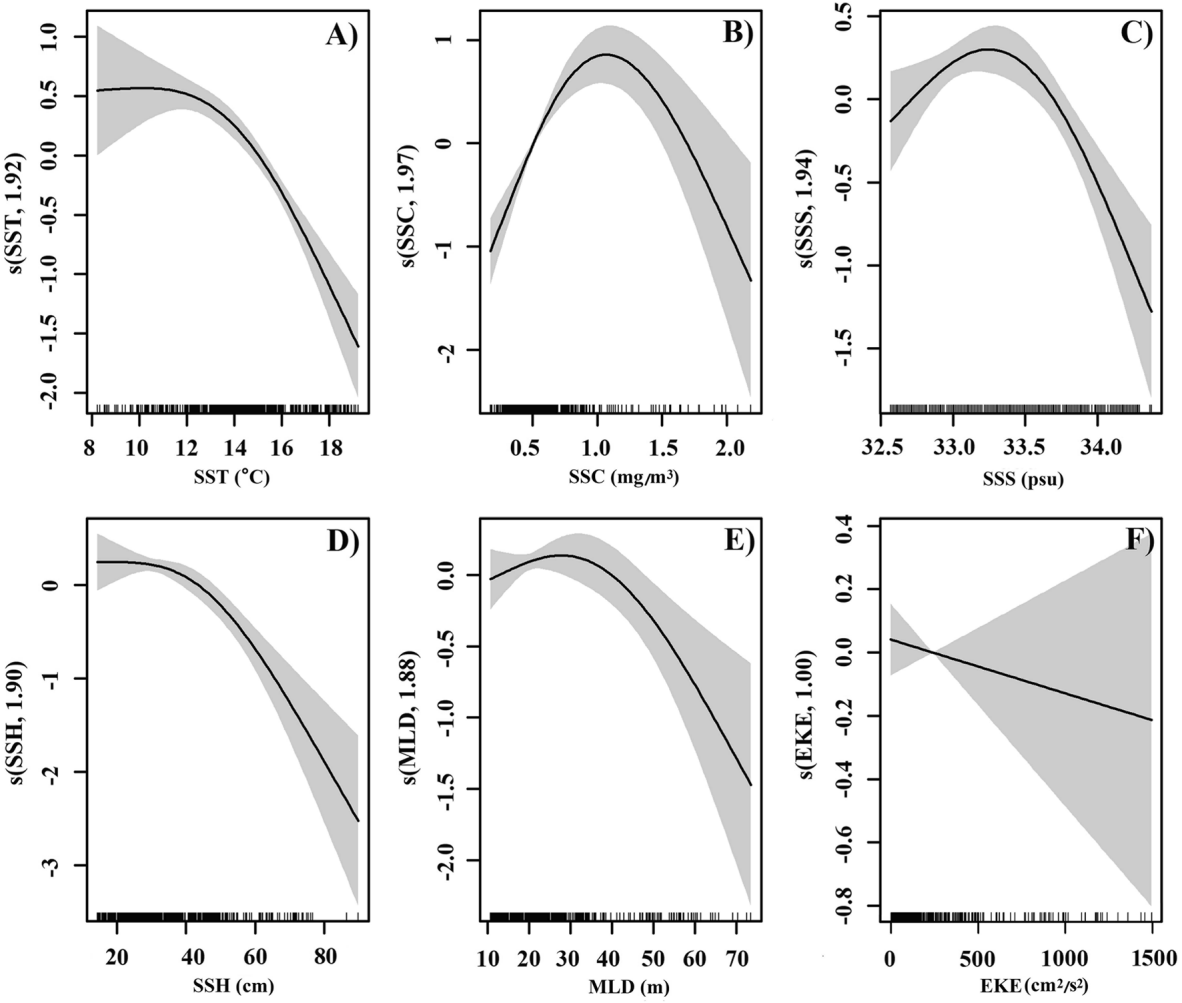
GAM-derived effects of the oceanographic variables on CPUE_1_, from models constructed for: (**A**) SST, (**B**) SSC, (**C**) SSS, (**D**) SSH (**E**) MLD, and (**F**) EKE. Rug plots on the horizontal axis represent observed data points, and the fitted function is shown by the solid line. The grey-shaded areas show the 95% confidence intervals.

In GAM analyses, the plot of partial residuals can be interpreted as the individual effect of each predictor variable on monthly CPUE_1_ (Fig. 8). The solid line shows the fitted GAM function, which describes the effect that a predictor variable has on the response variable. From 8 to 12 °C, SST showed a smooth trend on monthly CPUE_1_ and a significant negative effect on monthly CPUE_1_ above 12 °C. A positive effect on monthly CPUE_1_ was observed for SSC at 0.2–1 mg/m^3^, but at 1–2 mg/m^3^, SSC had a sharp negative effect on monthly CPUE_1_; SSS had a positive effect on monthly CPUE_1_ at < 33.3 psu, but a negative effect on monthly CPUE_1_ at > 33.3 psu; SSH had a negative effect on monthly CPUE_1_ across the whole observation range; MLD had a positive effect on monthly CPUE_1_ at values below 30 m, but a negative effect at 30–70 m; EKE had a linear negative effect on monthly CPUE_1_ with large confidence intervals for relatively large values of EKE.

#### Currents and eddies

**Figure 9:**
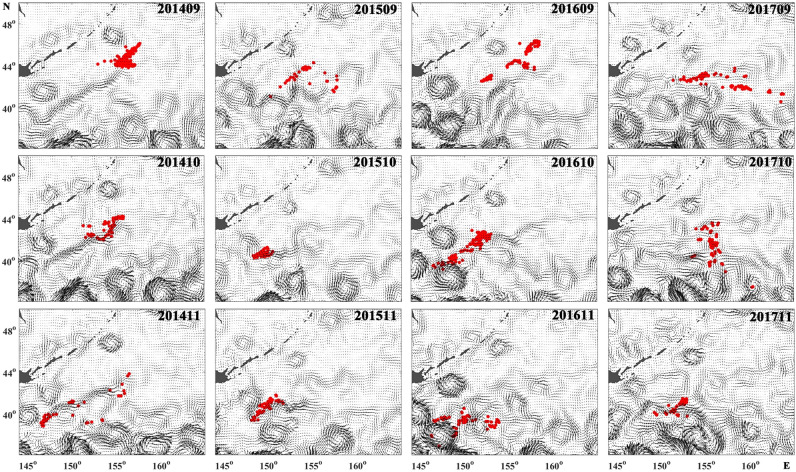
Spatial distribution of Pacific saury fishing locations superimposed on monthly geostrophic currents in September–November between 2014 and 2017. Fishing locations are shown as red dots. Maps were created using MATLAB R2016a sofware by MathWorks (https://ww2.mathworks.cn/products/matlab.html).

**Figure 10:**
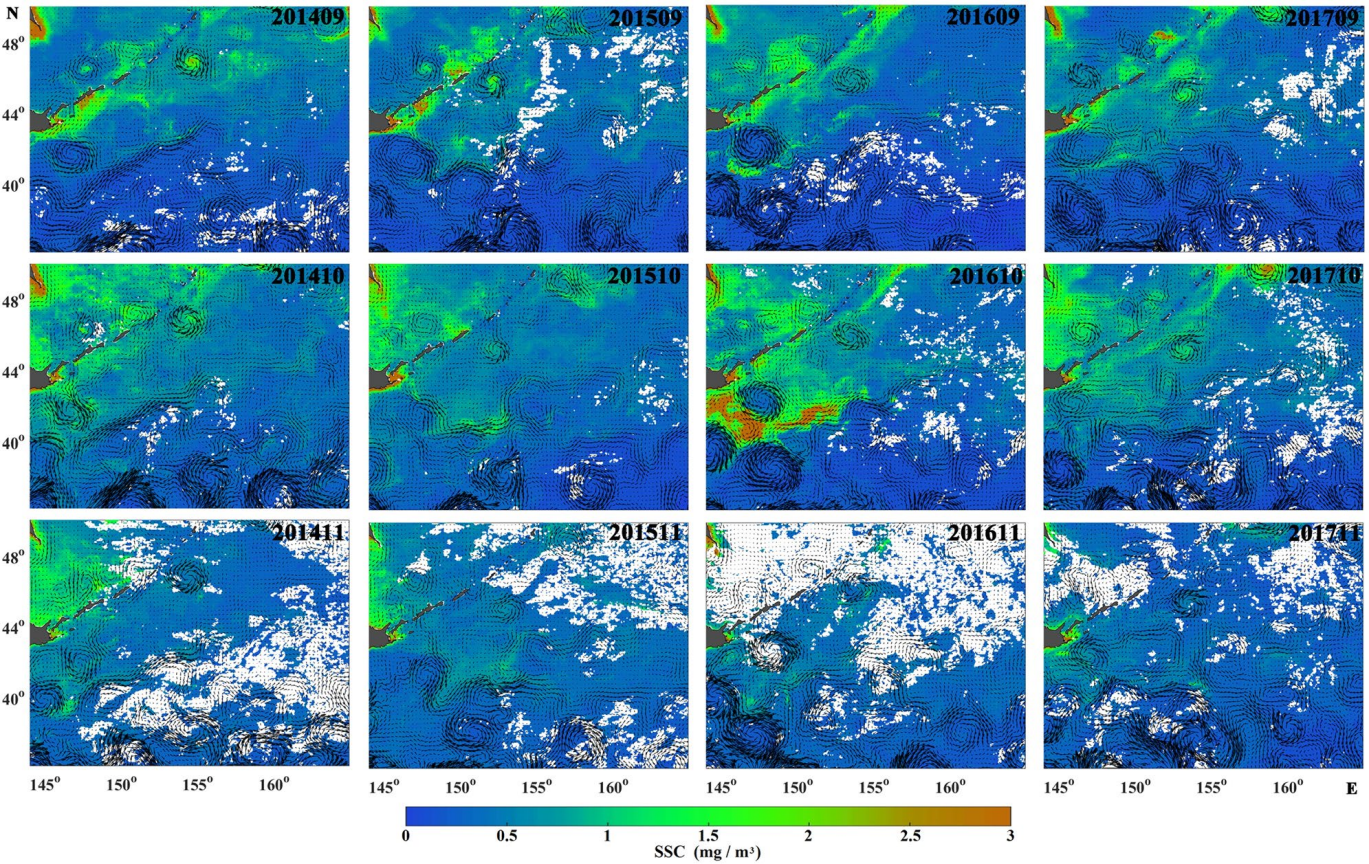
Relationships between SSC and geostrophic currents in September–November between 2014 and 2017. Maps were created using MATLAB R2016a sofware by MathWorks (https://ww2.mathworks.cn/products/matlab.html).

In order to more directly show the relationship between currents and the distribution of fishing locations, the fishing locations were superimposed on the geostrophic currents (Fig. 9). As the fishing season progressed, the positions of the fishing grounds moved gradually southwest and reached the Offshore Oyashio current and Kuroshio–Oyashio Transition Zone in September–November. The fishing locations were usually distributed along the Offshore Oyashio current and in its meander when the Oyashio was strong, such as in 2014 and 2015. There are many meso-scale eddies in northwestern Pacific, especially in the Kuroshio–Oyashio Transition Zone. When the Pacific saury crossed the Offshore Oyashio current to the Kuroshio–Oyashio Transition Zone, fishing was likely to occur at the peripheries of those eddies, such as in 2016 (Fig. 9). The distribution of SSC is greatly affected by ocean currents (Fig. 10). The Oyashio and its meander showed higher SSC concentrations than the surrounding region, whereas the SSC concentration was higher at the peripheries of clockwise eddies than at their centres.

### The yearly variation in migration routes of Pacific saury caused by the Oyashio intrusion area

**Figure 11:**
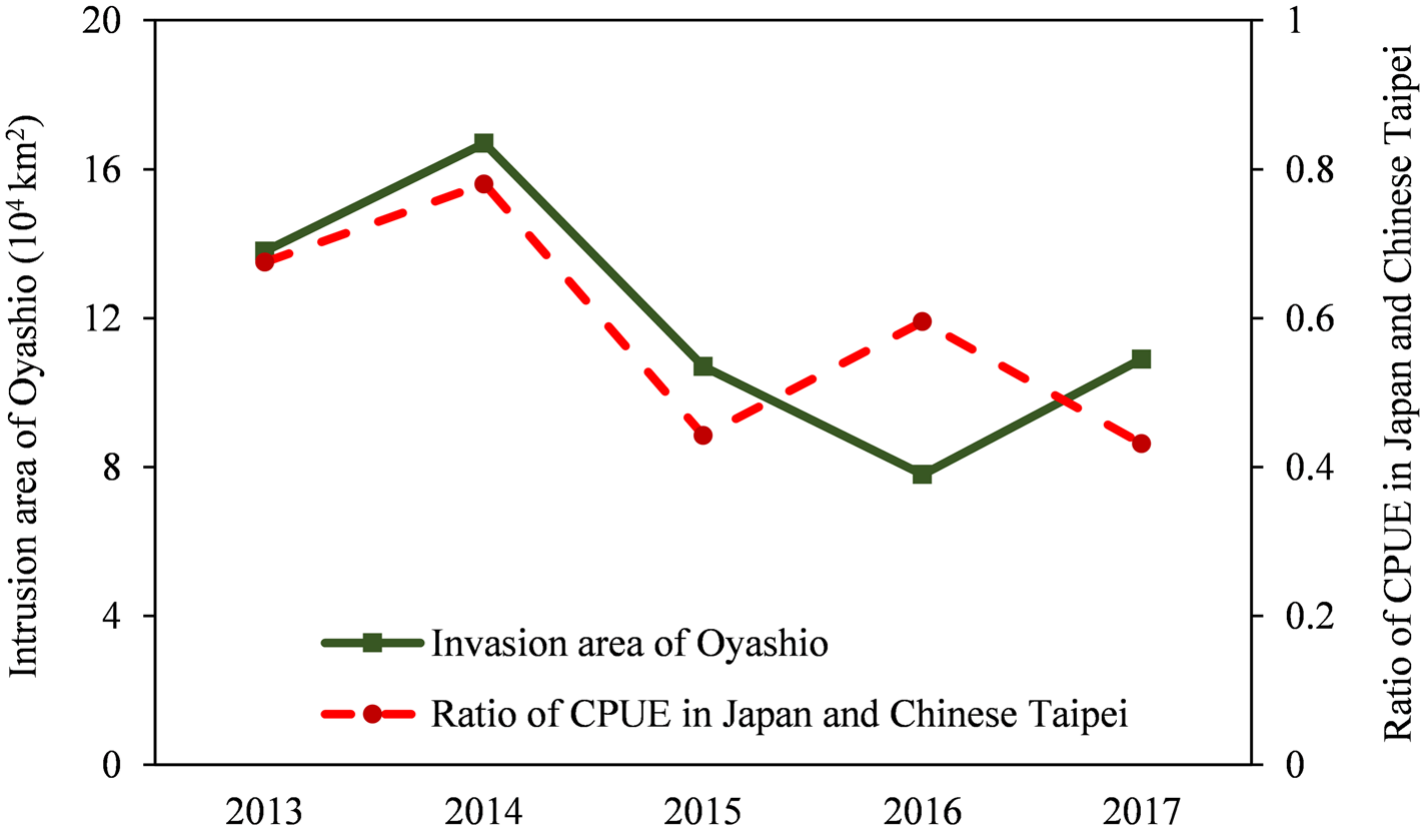
Variations in the intrusion area of the Oyashio and the relative abundance variation index.

The variations in the intrusion area of the Oyashio were similar to relative abundance variation index between Japan and Chinese Taipei, except in 2016 (Fig. 11). This indicates that the increase in the intrusion area of the Oyashio usually pushed the migration of Pacific saury to the Japanese coastal region. In contrast, when the intrusion area of the Oyashio was reduced, more fish migrated to the high-sea fishing ground. The intrusion area of the Oyashio was smallest in 2016 when the CPUE of Japan was highest, which may have been caused by the abnormal increase in SSC east of Hokkaido in October 2016 (Fig. 10).

## Discussion

### Catch better reflects the distribution of Pacific saury than CPUE

We used both monthly CPUE_1_ and CPUE_2_ as response variables in the gradient forests analysis, where monthly CPUE_1_ reflects the average catch, while monthly CPUE_2_ reflects the fishing efficiency. The results showed that the environmental factors explained the variations in monthly CPUE_1_ better than those in monthly CPUE_2_ (Fig. 5). It has been recognized that monthly CPUE_2_ can not accurately reflect fish abundance in some fisheries^26^. Previous studies have dealt with the general theory and concepts that relate CPUE to abundance, but the spatial component of this relationship has largely been ignored. Pacific saury fishing vessels have advanced fish-finding equipment and are connected effectively with one another, which causes a biased distribution of fishing vessels. Competition between fishing vessels occurs in high-abundance areas, where the total production is high but with only medium monthly CPUE_2_. Therefore, catch has been used as the abundance index in developing habitat suitability index models for the Pacific saury^27^.

### Effects of oceanographic environment on the distribution of Pacific saury

The distributions of oceanic fish species depend on several oceanographic factors, including but not limited to temperature, salinity, and nutrient availability, which are related to complex dynamic processes, such as MLD, current, and eddies^21,23,26,28^. In this study, we used a gradient forest analysis to evaluate the importance of each environmental factor to the distribution of Pacific saury. The gradient forest analysis directly reflects the importance of each factor in explaining the dependent variable. Our results show that SST is the most important factor affecting the monthly CPUE_1_ of Pacific saury, followed by EKE, with SSC as the least important factor.

Growth, feeding, distribution, and migratory patterns of Pacific saury are all affected by SST^29–31^. The geographic distributions and abundances of Pacific saury fishing stocks in the northwestern Pacific are strongly linked to sea temperatures^9,22^. From September to November, the Pacific saury migrates southwest with the gradual decline in SST in the northwestern Pacific. Tseng et al.^20^ reported that the optimal SST range for Pacific saury is 12–18.5 °C, with significant monthly variability^20^. In the present study, Pacific saury was distributed in an SST range of 8.1–20.6 °C, with optimal SST ranges of 14–16 °C in September, 13–16 °C in October, and 11–16 °C in November (Fig. 3). The median of the optimal SST for Pacific saury decreased from September to November, indicating that the SST in the fishing grounds declines faster than the migration speed of Pacific saury. In this study, Pacific saury fishing sites were mainly distributed in areas with SSTs around 15 °C (Fig. 2), which is consistent with previous reports^9,20^. However, SST showed a significant negative effect on monthly CPUE_1_ in the GAM (Fig. 8), indicating that the Pacific saury is likely aggregate in cool waters at least in 2014–2017.

The migration of Pacific saury is thought to be driven by the requirement for suitable water temperatures for spawning and the need for optimal access to food resources^18,19,25^. SSC, a proxy of the phytoplankton biomass, provides valuable information about the trophic interactions in marine ecosystems, and is considered an important factor in the formation of fishing grounds^13,27^. Tseng et al.^32^ demonstrated that a high CPUE for Pacific saury occurred when SSC ranged from 0.4 to 0.6 mg/m^3^, which is similar to our results^32^ (Fig. 4). Monthly CPUE_1_ showed a dome-shaped response to SSC, with the peak at 1 mg/m^3^, in the GAM analysis. However, the results of gradient forest analysis showed that SSC was the least important of the six factors examined (Fig. 6). In fact, Pacific saury usually ranges from the surface to 230 m and probably cannot tolerate lower temperatures encountered below the thermocline^20^. Therefore, the distribution of Pacific saury is affected by the surface environment and by deeper structures in the ocean. Mixed-layer processes are important for biological processes, and a deeper MLD may entrain more nutrients to the upper layer and provide a more vertically uniform water environment^33,34^. Yasuda and Watanabe^13^ suggested that the food supply for Pacific saury larvae is better at shallow MLDs and worse at deep MLDs^13^. In the present study, most fishing locations were located at MLDs below 35 m and the monthly CPUE_1_ of Pacific saury was higher at shallow MLDs, with peak monthly CPUE_1_ at an MLD of 30 m. This result can be explained by the particular operations of Pacific saury fishery. Pacific saury is caught with stick-held dip nets with attracting lamps, at fishing depths of 0–40 m^35^. The increase in MLD makes more food available for the Pacific saury, but it also leads to a deeper and more dispersed vertical distribution of the fish. Therefore, an appropriate MLD improves both the aggregation of the fish and its commercial capture.

The fishery data we used are from Chinese fishing vessels, which were not allowed to operate in the EEZ of Japan and Russia. Historical fisheries dada shows that waters along EEZ of Japan and Russia were not the important fishing ground of Pacific saury with high catch^1^. As many studies have demonstrated the importance of SST and Oyashio Current on the distribution of Pacific saury, SST and EKE will still be the most important environmental factors in the gradient forests analysis even if some data in the EEZ was added in the model^9,15,16,21^. However, the order of importance of SSC, SSS, SSH and MLD in gradient forests analysis and the corresponding trend of environmental factors to CPUE fitted by GAM may change slightly with adding the more data.

### Effects of Oyashio on the distribution and migration route of Pacific saury

The effects of ocean current and eddies on the coastal fishing grounds of Pacific saury have been determined in previous studies^15,25,36,37^. Meanwhile, mesoscale oceanographic features have been proved to be important factors in establishing habitat models for Pacific saury^38^. In this study, we used SSH and EKE to investigate the relationship between the distribution of Pacific saury and ocean dynamics in high-sea fishing grounds. The differences in SSH were attributed to ocean current and eddies. In the northern hemisphere, clockwise eddies have higher SSH in their interiors and lower SSH at their peripheries^28^. Our results show that EKE was the second most important predictor for Pacific saury. The fishing locations were often distributed in the Offshore Oyashio current in the Oyashio region and at the peripheries of clockwise eddies in the Kuroshio–Oyashio Transition Zone in October and November. The concentration of fishing locations along the border between the Russian/Japanese EEZs and high seas is due to Offshore Oyashio current, not because higher abundance of Pacific saury in the EEZs. Therefore, in 2017, when the Offshore Oyashio current was weak, the distribution of fishing locations appears far east of the EEZs and is more dispersed (Fig. 9).

Convergent oceanographic structures (i.e., fronts) can cause the aggregation of objects drifting on the ocean surface, and enhanced biological activity in these areas may improve the probability of highly migratory species encountering favourable feeding opportunities^21,28,39,40^. The Oyashio moves through the Four Islands and the eastern Hokkaido coastal region, where the water is rich in nutrients^41^. The high abundance of Pacific saury in coastal waters is probably related to the southward extension of the Oyashio Current, which correspond to lower SST and SSC in coastal areas^15,32,42^. The Oyashio carry high-nutrient water to the high-sea fishing ground. When the current turn or develop a meander, the nutrient-rich water concentrates in those areas, forming favourable feeding grounds for Pacific saury. There are many clockwise eddies in the northwestern Pacific, particularly in the Kuroshio–Oyashio Transition Zone. These clockwise eddies are convergent (downwelling) in their interiors but have zones of divergence (upwelling) near their peripheries^43^. This is also a favourable pattern for trophic succession, and the nutrient-enriched newly upwelled waters produced near the eddy edges are carried towards the convergent eddy interiors.

Our analysis of the variations in the intrusion area of the Oyashio and fisheries data for Pacific saury in Japan and Chinese Taipei mainly attributes the variations in the ratio of CPUE in the Japanese coastal and high-sea fishing grounds to the intrusion area of the Oyashio (Fig. 11). An increase in the intrusion area of the Oyashio causes more Pacific saury to migrate to the EEZ of Japan. Kuroda and Yokouch^19^ also reported that meso-scale eddies frequently occur near the Hokkaido coast, which prevent the southward intrusion of the Oyashio and reduce the Pacific saury catch in Japan^16^. Therefore, the route and intensity of the Oyashio dictates the migration route of Pacific saury and changes of the distribution of Pacific saury in the Japanese EEZ and high-sea fishing grounds. Consequently, after 2014, the significantly lower catch of Pacific saury in Japan was caused not only by reduced recruitment in the Kuroshio region, but also by the smaller intrusion area of the Oyashio^10^. Meanwhile, we try to extended the time series of both relative abundance variation index and Oyashio intrusion area, however, there was no significant correlation between the two sets of data. This may be due to the fact that long-term variations in relative abundance variation index are also driven by other factors. In addition to Oyashio, Kuroshio and Kuroshio-Extension may also affect the migration route of Pacific saury. Since the spawning ground of Pacific saury is located in the Kuroshio region, the strength of the Kuroshio and the extent of Kuroshio Meandering may affect the transport of eggs and juveniles, which in turn affects the distribution and migration route of Pacific saury in the fishing grounds^25,44,45^.

## Conclusions

We analysed the relationships between the oceanographic environment and the distribution of Pacific saury in the high-sea fishing ground in autumn, and our conclusions can be summarized as follows. Environmental factors predict monthly CPUE_1_ better than they predict monthly CPUE_2_, with SST as the most important predictor of monthly CPUE_1_, followed sequentially by EKE, SSS, SSH, MLD, and SSC. Monthly CPUE_1_ was negatively correlated with SST, SSH, and EKE, whereas monthly CPUE_1_ showed dome-shaped responses to SSC, SSS, and MLD, with peaks at 1 mg/m^3^, 33.3 psu, and 30 m, respectively. The Pacific saury is likely to localize to Offshore Oyashio current and the peripheries of clockwise eddies, where SSC is highest in October and November. The variations in the route and intensity of the Oyashio significantly affect the migration route of Pacific saury, and therefore the variations in its abundance in the EEZ of Japan and high-sea fishing grounds. Our research provides a theoretical basis for establishing an accurate forecasting system for high-sea Pacific saury fishing grounds.

## Methods

### Fisheries data

The fisheries data for Pacific saury used in this study were provided by Qingdao Zhongtai Oceanic Fishery Co., Ltd, China (one of the Chinese major fishing companies). The numbers of the fishing vessels involved from 2014 to 2017 were 6, 3, 8, and 4, respectively. The fishery data include daily geo-referenced fishing locations (latitude and longitude) and the catches in tonnes for each fishing vessel from September to November in 2014–2017. All the fishing vessels were the same size, similarly equipped, and used the same fishing method and nets. The fishing vessels were 78 m in length and 1655 tonnes in weight, and used a stick-held dip net. The size of the net was 38.3 × 41.7 m^2^ and the minimum mesh size of the net was 1.5 × 1.5 cm^2^.

The monthly CPUE_1_ (ton/vessel) and CPUE_2_ (ton/day) in each 0.25° grid (latitude × longitude) in 2014–2017 were calculated to analyse the relationships between the Pacific saury and the oceanographic environment. Because the number of fishing vessels involved differed in different years, we defined the monthly CPUE_1_ of the grids as the monthly total catch in grids divided by the number of fishing vessels in the month. We defined the monthly CPUE_2_ in the grids as the catch per fishing day of all the fishing vessels. Monthly CPUE_1_ reflects the average catch in the grid, while monthly CPUE_2_ reflects the fishing efficiency in the grid.

To investigate the influence of changes in the Oyashio on the distribution of Pacific saury, the relationships between the intrusion area of the Oyashio in the Japanese coastal region and the CPUEs of Pacific saury in the coastal and high-sea fishing grounds were analysed respectively. By considering the stability of long fisheries and the maturity of fishing techniques, we use data from Japan and Chinese Taipei to represent the coastal and high-sea fishing ground respectively. The Japanese and Chinese Taipei fisheries data, including the fishing days and total catch from 2013 to 2017, were obtained from North Pacific Fishery Commission (NPFC. https://www.npfc.int/summary-footprint-pacific-sauryfisheries). The average annual CPUEs (ton/day) of the Japanese fishing vessels (fishing in the EEZ of Japan) and the Chinese Taipei fishing vessels (fishing in the high seas) were calculated as the total catch divided by the total fishing days, to represent the abundance of Pacific saury in the Japanese coastal and high-sea fishing grounds, respectively. The average tonnage of the Japanese fishing vessels was about 80 tonnes and that of Chinese Taipei vessels was about 1100 tonnes (https://www.npfc.int/compliance/vessels). Because the Chinese Taipei fishing vessels were significantly larger than the Japanese vessels, the fishing efficiencies of Chinese Taipei and Japan differed considerably. To exclude the influence of unstandardized CPUE, we define a relative abundance variation index calculated by the ratio of CPUE in Japan to CPUE in Chinese Taipei. The value of the ratio has no practical meaning, but the annual variations in the ratio were used to reflect the variations in the relative abundance of Pacific saury in the coastal and high-sea fishing grounds. The ratio was also used to remove the effect of the annual variations in the total Pacific saury resources caused by climate change on the absolute values of CPUE.

### Environmental data

The monthly oceanographic environmental data in September–November between 2014 and 2017 were compiled for SST, SSC, SSS, SSH, MLD, and EKE.

The monthly SST and SSC, with a spatial resolution of 0.05°, were obtained from the Ocean Colour website (http://oceanwatch.pifsc.noaa.gov/thredds/catalog.html) Geostationary Operational Environmental Satellites (GOES) dataset and Visible Infrared Imaging Radiometer Suite dataset, respectively. The spatial resolution of SST and SSC were averaged to a 0.25° grid, corresponding to the spatial resolution of the fisheries data. The monthly SSS and MLD, with a spatial resolution of 0.25°, were obtained from the Copernicus Marine Environment Monitoring Service Global ARMOR3D L4 dataset (http://marine.copernicus.eu/ ). The daily SSH and geostrophic currents (u and v components), with a spatial resolution of 0.25°, were derived from the AVISO Delayed-Time Reference global Mean Sea-Level Anomaly product, and were converted to monthly averages. To represent the energy of sea surface current, we used EKE, calculated from u and v with Eq. (1)^46^.

1 $${\text{EKE}} = 1/2\left( {{\text{u}}^{2} + {\text{v}}^{2} } \right)$$

where u and v represent the meridional and zonal components of geostrophic current respectively.

The average annual data on the intrusion area of the Oyashio into the Japanese coastal region from 2013 to 2017 were obtained from the Japan Meteorological Agency (http://www.data.jma.go.jp/gmd/kaiyou/shindan/index_curr.html). The intrusion area of the Oyashio was defined as the area with temperatures < 5 °C at 100 m depth south of 43° N and west of 148° E^47,48^.

### Gradient forests analysis

In order to quantitatively analyse the importance of environmental factors to the distribution of Pacific saury we used the gradient forest machine learning approach (R package gradient forest, R Core Team, 2012). The gradient forest method is built upon random forests (regression trees that partition the response variable into two groups at a specific split value for each predictor *p* to maximize homogeneity) which are used to capture complex relationships between potentially correlated predictors and multiple response variables by integrating individual random forest analyses over the different response variables^49^. An independent bootstrap sample of the data is used to fit each tree, and the data not selected in the bootstrap sample (i.e., out-of-bag [OOB] data) are used to provide a cross-validated estimate of the generalization error. Together with other measures, gradient forests provide the goodness-of-fit ${(R}_{f}^{2}$) value for each response variable *f*, and the accuracy importance $({I}_{fp}$) of the predictor *p*. The importance of a split value along a predictor gradient reflects the relative change in the response variable. In this study, six environmental factors (SST, SSC, SSS, SSH, MLD, and EKE) were used as predictors, and monthly CPUE_1_ and CPUE_2_ were used as the response variables. Total 500 individual trees were built in the model. We ran the gradient forest 1000 times to obtain the mean and standard deviation (*sd*) of ${R}_{f}^{2}$. The run with the highest overall performance (*R*^2^) was then used to derive ${I}_{fp}$ and calculate the mean importance and the cumulative importance distribution of environmental factors.

### Generalized additive model (GAM)

As the gradient forests analysis cannot show the response curve of the CPUE with the change of environmental factors, a GAM^50^ was used to model the influence of environmental factors on the trends of CPUE. To avoid collinearity and for consistency, CPUE were modelled as smoothing functions for a single environmental variable. The effective degrees of freedom (representing the level of non-linearity) were restricted to a maximum of three to avoid over-fitting and to limit the driver–response relationships to a biologically realistic set of shapes (linear, dome-shaped, or sigmoidal) in the model^51,52^. A model of the form shown in Eq. (2) was applied:

2 $${\text{Log}}\;({\text{CPUE}}) \sim {\text{s}}\left( f \right) + {\text{Month}}$$

where *f* represents the independent continuous variable (SST, SSC, SSS, SSH, MLD, or EKE); and *Month* are the categorical variables. The CPUE data were logarithmically transformed to ensure that they were normally distributed. After the data were logarithmically transformed, both monthly CPUE_1_ and CPUE_2_ were normally distributed, indicating that our decision to transform the data was appropriate (Fig. S2).

### Ethics declarations

This study did not involve experiments on humans or animals.

## Supplementary Information

Supplementary Information.
