# Supplementary material for: Effects of oceanographic environment on the distribution and migration of Pacific saury (Cololabis saira) during main fishing season
Source: Sci Rep. 2022 Aug 9;12:13585. doi: 10.1038/s41598-022-17786-9 (PMC9363446; doi:10.1038/s41598-022-17786-9)
Supplement: Supplementary file 1 — Supplementary Information. [file 41598_2022_17786_MOESM1_ESM.pdf]

Supplement

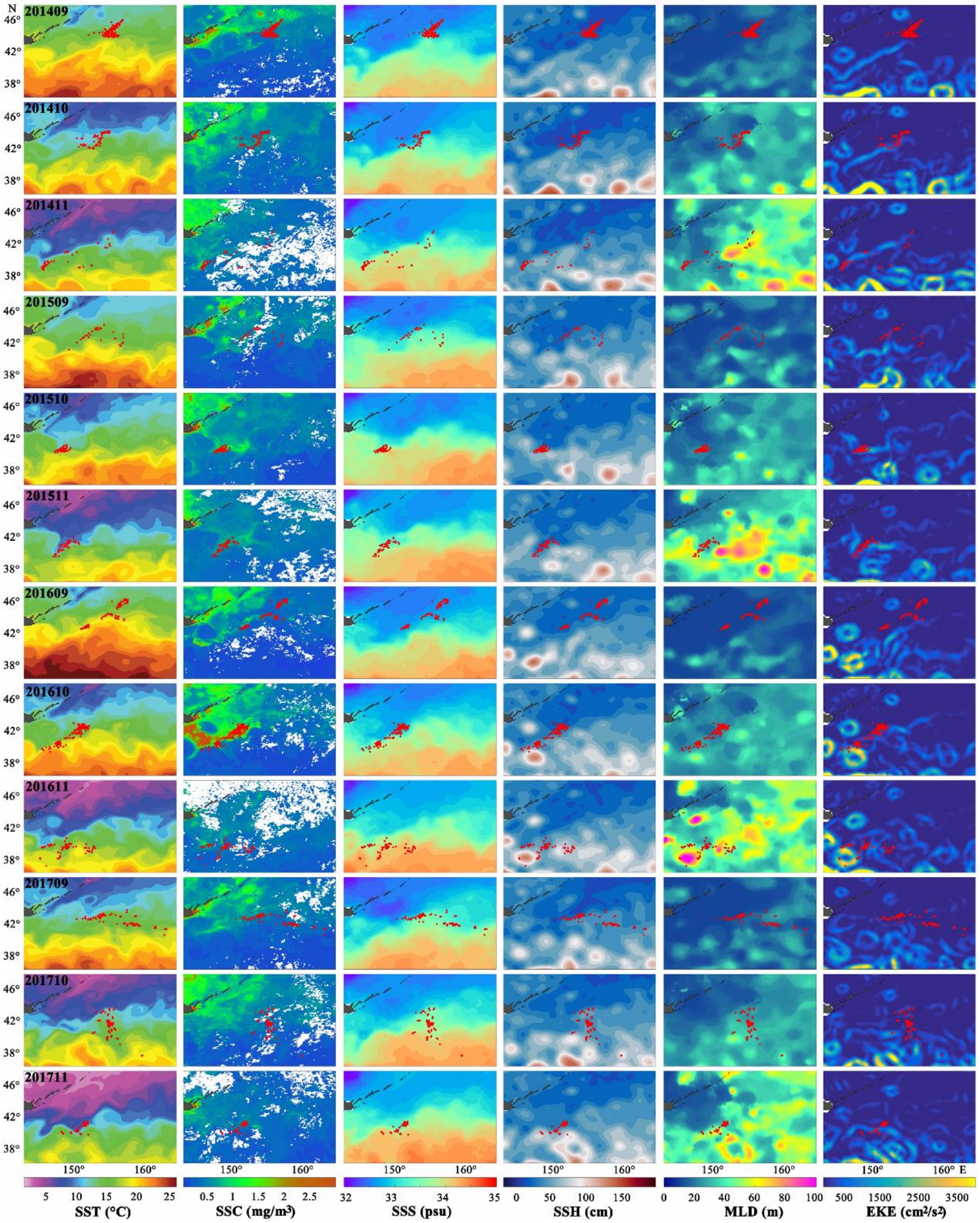

Fig. S1 Spatial distribution of Pacific saury fishing locations superimposed on monthly images of each of the six environmental variables (SST, SSC, SSS, SSH, MLD, and EKE) from September to November in 2014–2017. Fishing locations are shown as red dots. Black text on the images in the first column represents the year and month of the corresponding row of images. Maps were created using MATLAB R2016a sofware by MathWorks (https://ww2.mathworks.cn/products/matlab.html)

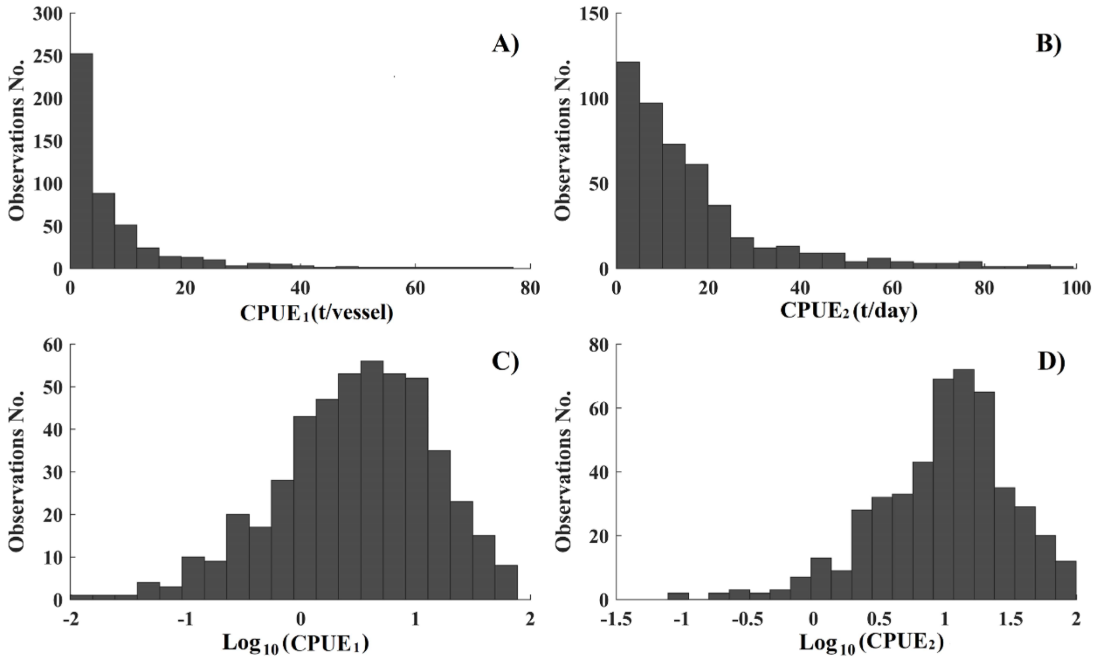

Fig. S2 Histograms of A) distribution of $CPUE_1$; and B) distribution of $CPUE_2$ of Pacific saury; and C) and D) distribution of log-transformed $CPUE_1$ and $CPUE_2$, respectively (which normalize the asymmetrical distributions).
